# Fabrication of deformable patient-specific AAA models by material casting techniques

**DOI:** 10.3389/fcvm.2023.1141623

**Published:** 2023-09-11

**Authors:** Maria Nicole Antonuccio, Emanuele Gasparotti, Francesco Bardi, Angelo Monteleone, Alexandre This, Laurence Rouet, Stéphane Avril, Simona Celi

**Affiliations:** ^^1^^ BioCardioLab, Bioengineering Unit - Heart Hospital, Fondazione Toscana “G. Monasterio”, Massa, Italy; ^^2^^ Philips Research Paris, Suresnes, France; ^^3^^ Mines Saint-Étienne, Université Jean Monnet, INSERM, Saint-Étienne, France; ^^4^^ Predisurge, Grande Usine Creative 2, Saint-Etienne, France; ^^5^^ Department of Radiology, Fondazione Toscana “G. Monasterio”, Massa, Italy

**Keywords:** abdominal aortic aneurysm, 3D printing, fused deposition modeling, lost molds casting, lost core casting

## Abstract

**Background:**

Abdominal Aortic Aneurysm (AAA) is a balloon-like dilatation that can be life-threatening if not treated. Fabricating patient-specific AAA models can be beneficial for *in-vitro* investigations of hemodynamics, as well as for pre-surgical planning and training, testing the effectiveness of different interventions, or developing new surgical procedures. The current direct additive manufacturing techniques cannot simultaneously ensure the flexibility and transparency of models required by some applications. Therefore, casting techniques are presented to overcome these limitations and make the manufactured models suitable for *in-vitro* hemodynamic investigations, such as particle image velocimetry (PIV) measurements or medical imaging.

**Methods:**

Two complex patient-specific AAA geometries were considered, and the related 3D models were fabricated through material casting. In particular, two casting approaches, i.e. *lost molds* and *lost core* casting, were investigated and tested to manufacture the deformable AAA models. The manufactured models were acquired by magnetic resonance, computed tomography (CT), ultrasound imaging, and PIV. In particular, CT scans were segmented to generate a volumetric reconstruction for each manufactured model that was compared to a reference model to assess the accuracy of the manufacturing process.

**Results:**

Both *lost molds* and *lost core* casting techniques were successful in the manufacturing of the models. The *lost molds* casting allowed a high-level surface finish in the final 3D model. In this first case, the average signed distance between the manufactured model and the reference was (−0.2±0.2) mm. However, this approach was more expensive and time-consuming. On the other hand, the *lost core* casting was more affordable and allowed the reuse of the external molds to fabricate multiple copies of the same AAA model. In this second case, the average signed distance between the manufactured model and the reference was (0.1±0.6) mm. However, the final model’s surface finish quality was poorer compared to the model obtained by *lost molds* casting as the sealing of the outer molds was not as firm as the other casting technique.

**Conclusions:**

Both *lost molds* and *lost core* casting techniques can be used for manufacturing patient-specific deformable AAA models suitable for hemodynamic investigations, including medical imaging and PIV.

## 1. Introduction

An abdominal aortic aneurysm (AAA) is a vascular disease that occurs when the arterial lumen dilates permanently and irreversibly, and the aortic diameter exceeds 30 mm (1). Currently, there are flaws in fully understanding the mechanisms of AAA initiation, development, and rupture, as well as in managing the treatment (2–5). In the biomedical field, extensive research has been performed throughout the last two decades to have insight into the AAA disease by exploiting both *in-silico* and *in-vitro* approaches as an effective alternative to animal experiments (6). Indeed, advances in medical imaging have enabled the acquisition of high-resolution images of the anatomy of interest, from which a 3D model can be accurately reconstructed and manufactured (7) for both planning and training (8–15).

In the context of functional investigations, the insertion of 3D models in a mock-circulatory loop (MCL) system provides a useful tool to reproduce and study the cardiovascular system at a patient-specific level in terms of geometry and hemodynamic conditions (16–18). Furthermore, imaging models inserted in MCL systems by means of magnetic resonance imaging (MRI) (19, 20), ultrasound (US) (21–23), and particle imaging velocimetry (PIV) (24) can deepen the knowledge of cardiovascular diseases, providing insights into the pathology conditions and progression, also in combination with *in-silico* studies (25, 26). However, each imaging modality requires specific features of the manufacturing material. Indeed, MRI applications require nonmagnetic materials, US imaging requires specific values of sound speed, acoustic attenuation, and echogenicity of the material (27–30), whereas models made of optically clear materials with the same refractive index as the blood-mimicking fluid are required for PIV applications (31). Either rigid or deformable models can be manufactured based on the application and purpose of the study. Glass and transparent resins are examples of materials used to manufacture rigid models, whereas rubberlike compounds, polyurethane, and silicone are examples of materials that can be used to fabricate deformable models.

Focusing on AAA 3D models, several works in the literature involved a variety of geometries, materials, manufacturing techniques, and applications. The geometry of AAA phantoms can be either idealized (32–35), which allows performing parametric studies and investigating complex hemodynamics in controlled conditions, or patient-specific (12, 36–38) to observe personalized morphological and hemodynamic features. Mock circulatory loops coupled with MRI systems were used to measure flows and wall shear stress (35), or assess temporal changes in intraluminal pressure and in the AAA sac (39). A model compatible with computerized tomography (CT) and US imaging was also manufactured and its utility was demonstrated during a simulated EVAR procedure (40). Finally, many studies performed PIV measurements to provide accurate quantification of the abnormal velocity fields in AAA by using rigid (32, 41, 42) or deformable silicone (34, 43–47) models.

In order to couple echogenicity, transparency, and deformability of the manufactured models, silicone-based materials have been proven to be the most suitable to manufacture complex anatomical models (30). In this regard, while the material is selected based on the type of application, the fabrication method is selected based on the material. Currently, silicone-based materials are challenging to directly print a model (48). Consequently, material casting techniques are preferable (49).

In material casting, silicone-based materials are poured into a mold, usually 3D-printed, which contains a hollow cavity of the desired shape where poured materials cure and solidify. Once the curing phase is complete, the mold is removed, thus obtaining the final manufactured model. In the case of hollow and thin-walled models, both inner (or *core*) and outer molds are required. Fabricating molds and deformable models is challenging in the case of complex geometries that require the use of sacrificial molds to fabricate the final model. In these complex cases, the core mold is always sacrificed, whereas the outer molds can be sacrificed (*lost molds* technique) or preserved (*lost core* technique) and reused for more castings.

However, despite a quite large production of these types of works, there is a significant lack of accurate information on the manufacturing of deformable phantoms, thus allowing the reproducibility and repeatability of the manufacturing process. This is particularly true in the case of 3D model fabrication through molding techniques, which are user-dependent and require multiple steps (3D printing, mold definition, mold assembly, material pouring, demolding) with different aspects to consider that can influence the success or the failure as well as the final results of the manufacturing process.

In this work, we present a detailed description of all the phases necessary to create complex (when undercuts are present) deformable CT-based AAA models to be inserted in a mock-circulatory loop system for image-based hemodynamic investigations. Both *lost molds* and *lost core* techniques are investigated and used to manufacture the AAA models. All design and fabrication phases are described, along with costs and equipment requirements, addressing the main advantages and limitations of the proposed methodologies.

## 2. Materials and methods

The manufacturing procedure to fabricate deformable models that mimic the compliant behavior of AAAs involved six main steps: (i) model creation; (ii) design of the molds; (iii) 3D-printing of the molds; (iv) surface treatment; (v) material casting; (vi) removal of the molds. Finally, the manufacturing accuracy was assessed. Each of these steps is detailed in the following sections.

### 2.1. Model creation

#### 2.1.1. Image dataset and segmentation

CT images of the thoracoabdominal anatomy of 85 and 78-year-old patients affected by AAA were used to create the 3D models. The datasets were provided by Rigshospitalet (Copenhagen, DK), which recruited patients from the Copenhagen Aneurysms Cohort (COACH), a cohort of patients with AAAs under surveillance with advanced ultrasound imaging and biochemistry (5).

**Figure 1:**
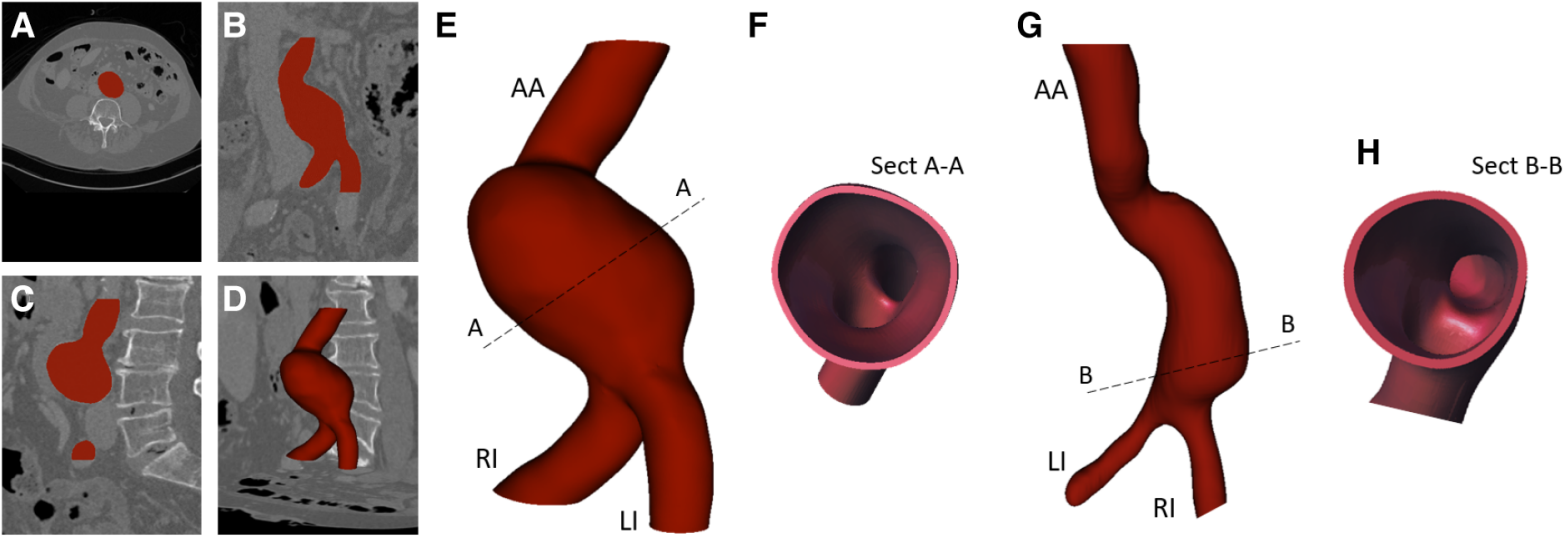
Example of image segmentation in axial (**A**), coronal (**B**), longitudinal view (**C**), and segmentation result (**D**); 3D solid models (**E**, **G**) with related cross-sections (Sect) (**F**, **H**).

The images were segmented and the volume of the vessels was reconstructed with the open-source software 3DSLICER (Slicer, NIH) (50) by following the approach previously described in Celi et al. (51). Figures 1A–C show an example of segmentation in the axial, coronal, and longitudinal planes. The segmented AAA geometry is shown in Figure 1D.

#### 2.1.2. AAA solid model generation

The segmented geometries were exported as stereo-lithography format (*stl*) files and imported into the solid modeling computer-aided design (CAD) software SpaceClaim (ANSYS, Inc.) where the associated solid models were generated by means of non-uniform rational basis-splines interpolation. The 3D solid models are shown in Figures 1E,G. Both models include the abdominal aortic inlet (AA), aneurysm bulge, Left Iliac artery (LI), and Right Iliac artery (RI), which are the two outlets. The solid models were then imported into the CAD software SOLIDWORKS (Dassault Systèmes S. A., Vélizy-Villacoublay, France). Here, outward offset values of 1.8 and 2 mm were applied to the AAA models, respectively, to fix the thickness as shown by the cross-sections in Figures 1F,H.

Among all those available, we chose these two patient-specific geometries because they are representative of challenges in the casting technique due to their shape complexity. The two models were manufactured by testing two techniques: a *lost molds* technique for the first AAA model, and a *lost core* technique for the second one, as described in the following sections. According to the casting technique, the two models were referred to as *lost molds model* (LMM) and *lost core model* (LCM).

### 2.2. Design of the molds

The core and outer molds were designed in SOLIDWORKS for both cases by following the same procedure as the designing of the molds does not depend on the casting technique.

#### 2.2.1. Core molds

**Figure 2:**
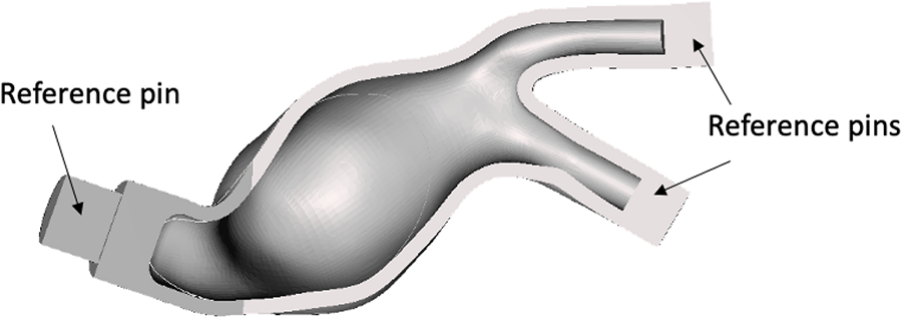
Example of core mold for the LMM with the associated reference pins.

Cores define the inner surface of the deformable models. As the first step, a hollow core was designed for each model by extracting the inner wall of the AAA solid models (in Figures 1E,G) and applying an inward offset value of 3 mm to define the thickness of the core. The LI and RI outlets and the AA inlets were closed and extruded 15 mm outward and inward, respectively, to obtain reference pins. Additionally, a 15 mm cylindrical pin was added to the inlets. Those pins allowed a firm alignment and a reference between the core and outer molds, thus maintaining the correct gap for material casting. Figure 2 shows the extrusion and reference pins related to the core of LMM.

#### 2.2.2. Outer molds

**Figure 3:**
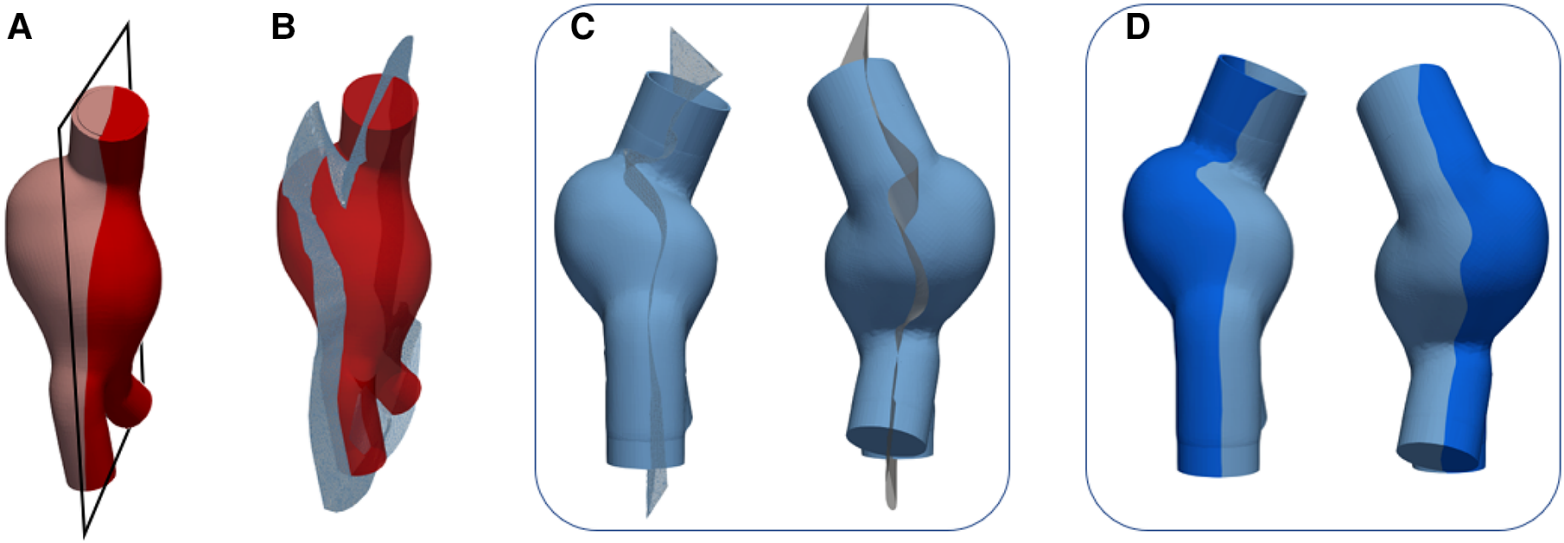
Outer mold splitting procedure for LMM: AAA solid model and plane used to define the pull direction (**A**) and parting surface (**B**); extrusion of the parting surface outward (**C**); outer molds after splitting (**D**).

**Figure 4:**
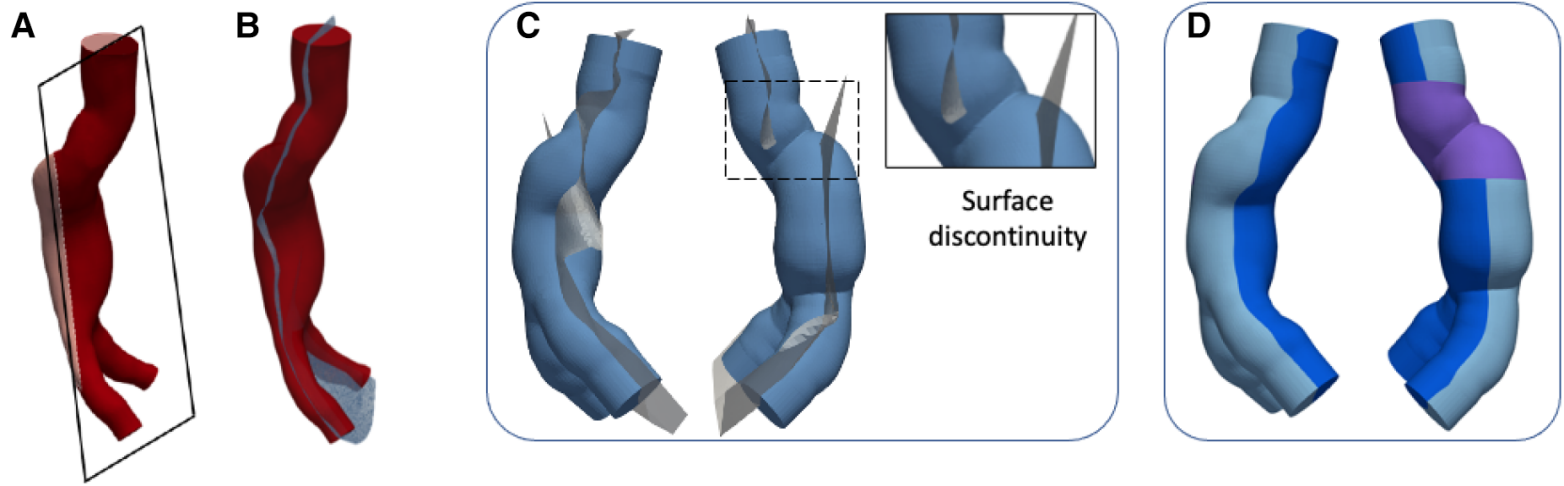
Outer mold splitting procedure for LCM: AAA solid model and plane used to define the pull direction (**A**) and parting surface (**B**); extrusion of the parting surface outward and detail of surface discontinuity (**C**); outer molds after splitting (**D**).

**Figure 5:**
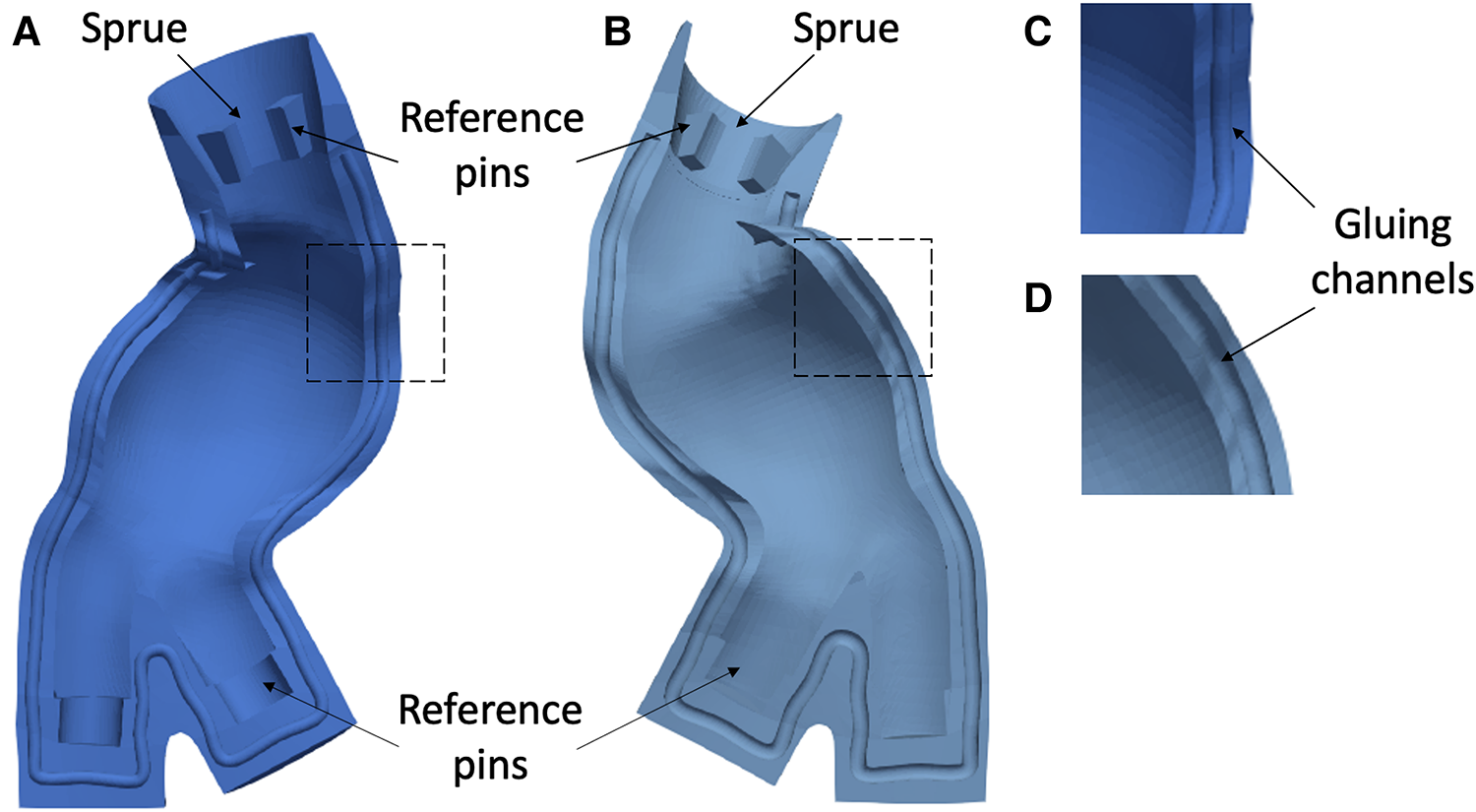
Example of outer molds for LMM and related sprue, reference pins (**A**, **B**), and gluing channels (**C**, **D**).

The outer molds define the outer surface of the manufacturing deformable models. For both LMM and LCM, outer molds were designed through a multi-steps strategy consisting of:

(1) *Creation of a monolithic external mold* - An outward offset value of 10 mm was applied to the outer surface of each AAA solid model (Figures 1E,G) after closing inlet and outlets. A monolithic outer mold, solid inside, was obtained. This mold can contain the core and AAA solid model.
(2) *Splitting of the outer molds* - Due to the complex anatomies, it was necessary to create a specific separation surface for each model to avoid undercuts and to facilitate the assembly. For this reason, a specific separation surface was created for each model and used as the trim tool.
  The AAA solid models (Figures 3A and 4A) were considered to generate the separation surface for each model. Furthermore, since non-conventional separation surfaces were employed instead of planes, we divided the outer mold appropriately to satisfy the requirement to assemble and dismantle the several components into which the outer mold would be separated, thereby ensuring the right gap between the core and outer molds. First, the pull directions and a draft angle of 4${}^{\circ }$ were selected to create a separation surface using the *parting line* and *parting surfaces* SOLIDWORKS tools. Specifically, the pull directions are the directions in which the two halves of a mold separate. In these cases, the pull directions were defined by the planes in Figures 3A and 4A. The so-obtained surfaces are shown in Figures 3B and 4B together with the AAA solid models. These surfaces were further extended outward since a wide enough surface is necessary to split the external mold (Figures 3C and 4C). We hence split the external mold by using those separating surfaces as the trim tools and obtaining the two parts of Figures 3D and 4D. Referring to the LCM only, there is a discontinuity in the separating surface (as shown in Figure 4C) that would have made the use of only two external molds impossible. As a result, this region was omitted from the two outer molds, and a third mold was created (in purple in Figure 4D), which is assembled with the other two orthogonally to the pull direction.
(3) *Subtraction of the core and AAA solid model from the outer molds* - From solid, the outer molds were made hollow by subtracting the core and AAA solid model from the outer molds. Therefore, the core and the outer molds could be assembled and disassembled successfully while ensuring the correct internal gap between them, which corresponds to 1.8 and 2 mm (thickness of models) for LCM and LMM, respectively.
(4) *Designing of sprues, reference pins for the core, and gluing channels*. We cut the top of the outer molds by removing material between two concentric circumferences so as to recreate a conical-shaped, 20-mm-high sprue (in Figures 5A,B for the LMM) that would facilitate the material pouring and prevent it from spilling out of the molds. We then added four 90${}^{\circ }$- equispaced pins on the inner surface of the sprue (as shown in Figures 5A,B for the LMM) that could serve as a support and reference for core placement. As a final step, we drew a spline that could internally follow the contour of one of the two outer molds. This spline defined the path to sweep a circular profile to create a groove on one outer mold and a complementary extrusion on the other as shown in detail in Figures 5C,D for the LMM. Radial and axial geometric tolerances were assigned to the outer molds in correspondence with the reference pins to ensure assembly.

### 2.3. 3D printing of the molds

Two casting techniques were tested: (i) *lost molds* casting, meaning that both core and outer molds were printed in polyvinyl alcohol (PVA), which is a water-soluble material, and dissolved once the casting material has cured; (ii) *lost core* casting, meaning that only the core was printed in PVA, whereas the outer molds were printed in acrylonitrile–butadiene–styrene (ABS) so that they may be reused to manufacture the same model several times. Core and outer molds were 3D printed with a fused deposition modeling (FDM) printer (A4v3, 3NTR Italy). The G-Code format files were generated with the dedicated software 3NTR SSI (3NTR, Italy).

#### 2.3.1. Lost molds

A PVA filament of 2.85 mm was used as the primary material for printing the molds and as support material. Rectilinear and honeycomb patterns were set for the support and infill, respectively. The support density was set to medium for the outer and to fine for the core molds. The main settings of PVA printing are detailed in Table 1.

**Table 1:** FDM parameters’ setting to print PVA and ABS.

Parameter	PVA	ABS
Printed components	LMM, LCM core	LCM outer molds
Layer height	0.15 mm	0.20 mm
Nozzle diameter	0.4 mm	0.4 mm
Nozzle temperature	245${}^{\circ }$C	240${}^{\circ }$C
Heated chamber temperature	40${}^{\circ }$C	80${}^{\circ }$C
Build plate temperature	70${}^{\circ }$C	105${}^{\circ }$C
Support material	PVA	SSU00
Support density	Medium/Fine	Medium
Infill Density	20%	16.7%
Material flow	100%	100%
Print speed	90%	100%
Number of contours	2	4

#### 2.3.2. Lost core

A PVA filament of 2.85 mm was used as the primary material for printing the core and as support material by setting a fine density. An ABS filament of 2.85 mm was used as the primary material for printing the outer molds, whereas the supports were printed in the high-impact polystyrene-based SSU00 (3NTR, Italy) material. Rectilinear patterns were set for the support as well as the infill. The main settings of ABS printing are detailed in Table 1.

### 2.4. Surface treatment

Prior to casting, supports were manually separated from the molds. The external surfaces of the cores and the internal surfaces of the outer molds (contact surfaces) were treated to eliminate surface roughness typical of FDM printing. Specifically, these surfaces were first carefully manually sanded with fine-grained sandpaper. Subsequently, we treated cores and outer molds depending on the *lost molds* or *lost core* casting technique.

#### 2.4.1. Lost molds

A solution of 15% (by weight) of low molecular weight PVA powder was prepared in distilled water at 120${}^{\circ }$C. This solution was stirred until the solute was completely dissolved, resulting in a transparent solution that was then evenly brushed twice onto the contact surfaces, alternating between brushing and air drying. This process made the contact surfaces smooth, ensuring a good finish to the final model. Finally, those surfaces were treated with a release agent (Mann, Macungie, Pennsylvania) to ensure that the casting material was completely released from the molds during molds’ dissolving. Once the contact surfaces were completely dry, we assembled the core, poured liquid vinyl glue into the gluing channels, and closed the outer mold with its counterpart, ensuring a firm press by means of ties. Acrylic glue was added at the joint edges of the outer molds, and some tape was affixed so as to prevent possible leakage of the casting material. The molds were then left in place for 12 h.

#### 2.4.2. Lost core

The contact surfaces of each mold were treated with clear acrylic paint (Ambro-Sol S.r.l., Italy) to make those surfaces hydrophobic thus avoiding the interaction between PVA and casting material. This interaction could alter the optical properties of the phantom. As for the *lost molds* casting, the surfaces adhering to the casting material were treated with a release agent (Mann, Macungie, Pennsylvania) to ensure that the casting material was completely released from the molds during external mold disassembling and core dissolving. Once the treated surfaces were completely dry, the molds were assembled and sealed by means of a two-component sealant specific for ABS (presto, European Aerosols, Wolvega, the Netherlands), applied to the joint edges of the outer molds so as to prevent possible leakage of the casting material. This specific type of sealant was employed since it can both provide a firm seal and be easily removed without destroying the molds as an acrylic glue would. Therefore, we waited until the sealant was completely dry before casting the material into the molds.

### 2.5. Material casting

Patient-specific AAA deformable models were made out of the two-component (silicone and curing agent) silicone material Sylgard 184 (Dow, Wiesbaden, Germany). Properties of the used material can be found in Table 2 (31).

**Table 2:** Mechanical and physical properties of the Sylgard 184 silicone.

Properties	Value
Young’s Modulus	1.25 MPa
Ultimate tensile strength	6.7 MPa
Shore hardness	43
Refractive Index	1.412
Density	1.05 g/cm${}^{3}$
Sound speed	1100 m/s

According to the instructions, we prepared the material with a 10:1 (silicone:curing agent) ratio. After stirring, the casting material was degassed in a vacuum chamber for 30 min at −0.8 bar to remove air bubbles resulting from the mixing of the two parts. Degassing cycles were repeated until the total removal of bubbles was achieved. Subsequently, the material was poured into the molds in several batches to prevent air bubbles being trapped trapped in thick layers. For this reason, several cycles of pouring/degassing at −0.8 bar for 30 min were alternated. We concluded the casting phase once the silicone had reached the upper edge of the inner mold, and double-checked that there were no visible air bubbles. The silicone was cured for 48 h at room temperature.

### 2.6. Removal of the molds

#### 2.6.1. Lost molds

Once the silicone has cured, the PVA components were dissolved by placing the entire assembly of molds in an ultrasonic tank filled with warm water and by securing the assembly to the bottom. 3 to 4 washing cycles were done, the first one about 1 h long at 60${}^{\circ }$C with ultrasound enabled to saturate the PVA faster. The glued parts of the molds were broken up by using small tools. The PVA dissolved leaving both a smooth lumen inside of the silicone and a smooth surface outside of the silicone. The LMM was finally left in cold water for a few hours.

#### 2.6.2. Lost core

Once the silicone has cured, the sealant for ABS was manually removed by sanding the zones of interest and then scratching the sealant using a scalpel. First, the purple component of Figure 4, which is orthogonal to the pull direction was disassembled. One out of two outer molds was also disassembled whereas the remaining outer mold, the core, and the silicone model were placed in an ultrasonic tank filled with warm water with enabled ultrasound. The outer mold was removed after about 20 min and the PVA core dissolved in about 4 h at 70${}^{\circ }$C. The LCM was finally left in cold water for a few hours.

### 2.7. Accuracy assessment

The accuracy of the manufactured models was assessed by comparing them and the original CAD models in terms of related meshes. The manufactured models were scanned with CT to generate image volumes comparable to the patient ones. The images acquired on each model were then segmented to reconstruct the so-called *manufactured* mesh (also including the wall thickness) that was compared to the mesh of the original CAD model (the so-called *reference* mesh). As the first step, the two meshes, which had a comparable number of vertices, were registered through iterative closest point algorithm (52) performing a rigid registration. Once registered, the signed distance was computed for each vertex of the compared mesh with respect to the reference mesh by using CloudCompare (v.2.13.alpha [GPL software], 2022) (53). The generated distance scalar field was hosted by the vertices of the compared mesh. The same procedure was performed for both LMM and LCM.

## 3. Results

### 3.1. 3D printing of the molds

**Figure 6:**
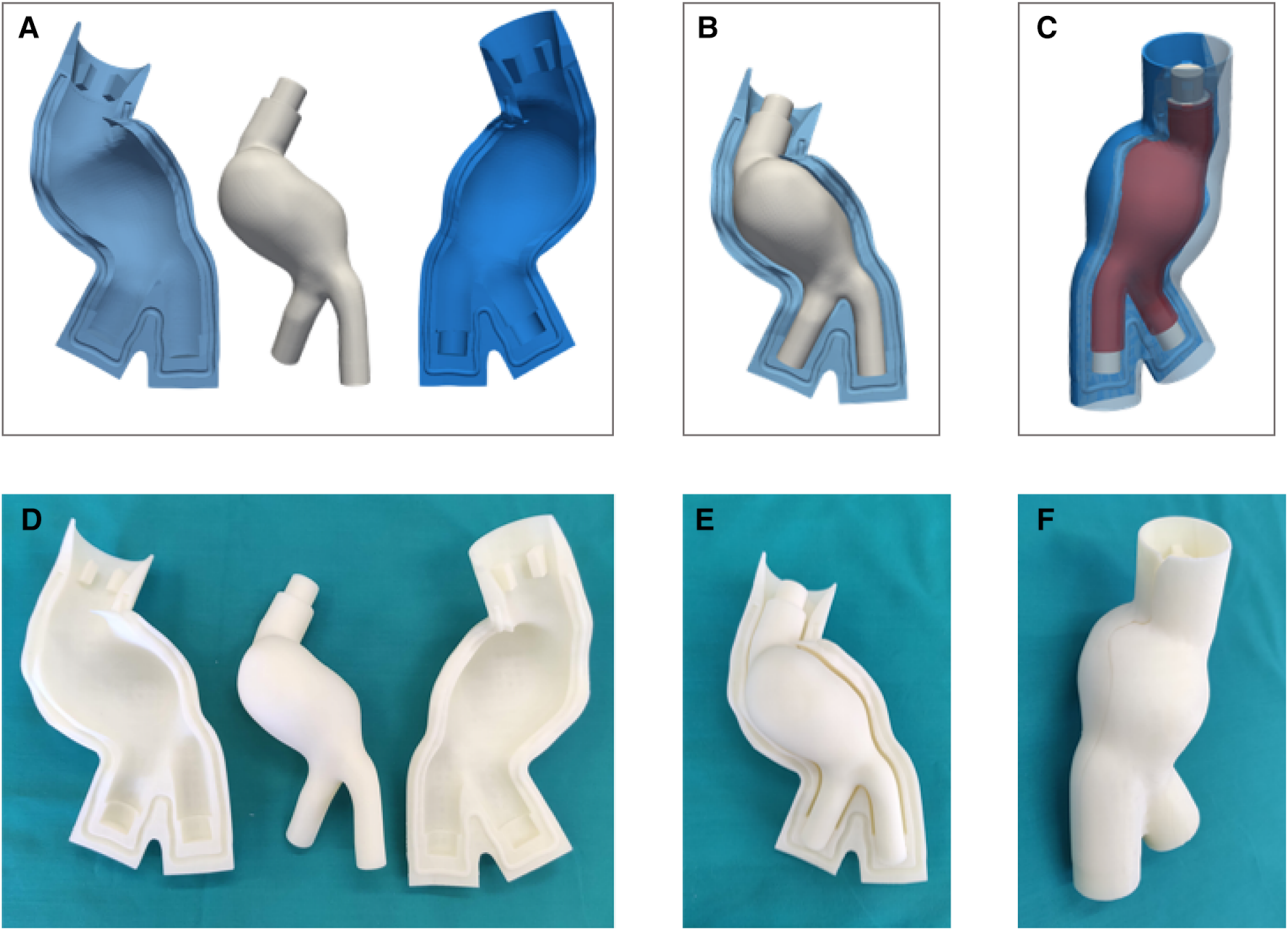
CAD representation (**A**–**C**) and 3D printing (**D**–**F**) of LMM molds.

**Figure 7:**
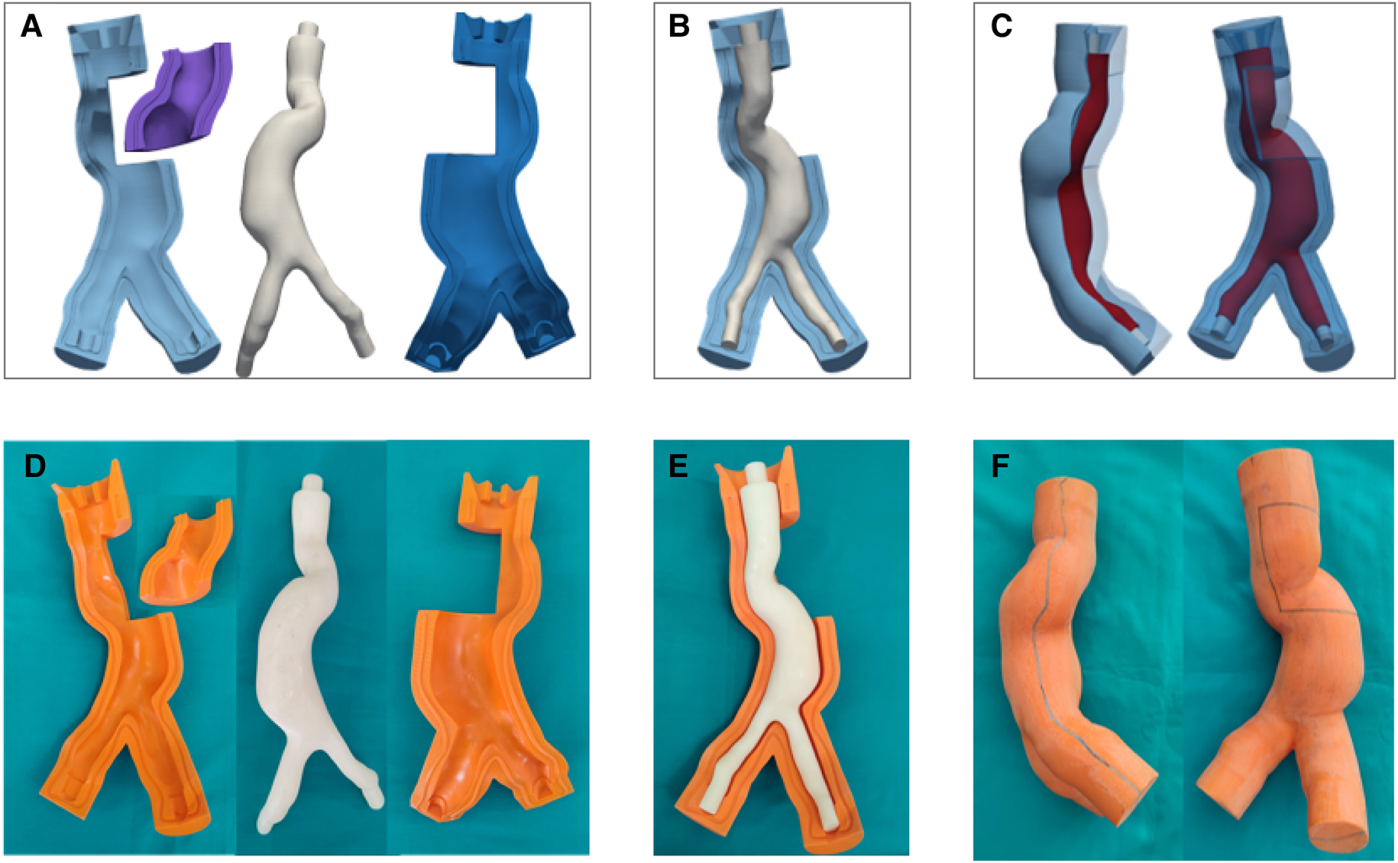
CAD representation (**A**–**C**) and 3D printing (**D**–**F**) of LCM molds.

Figures 6A–C and 7A–C show the CAD representation of the several components designed to fabricate the LMM and the LCM, respectively. Here, the whole assemblies, which also include the AAA solid models (in red in Figures 6C and 7C), are also given. Symmetrically, Figures 6D–F and 7D–F show the results of the mold printing via FDM for the LMM and LCM, respectively. The FDM printer accurately created outer and core molds, coherently with the original design. In particular, all the complexities of these patient-specific geometries were reproduced. The core and outer molds were easy to assemble, achieving a perfect closure while maintaining the correct inner gap into which the casting material would later creep as shown in Figures 6E and 7E. The sealing of the outer molds using PVA and acrylic glue or the sealant specific for ABS is shown in Figures 6F and 7F for the *lost molds* and the *lost core* casting techniques, respectively.

### 3.2. Material casting and cost analysis

**Figure 8:**
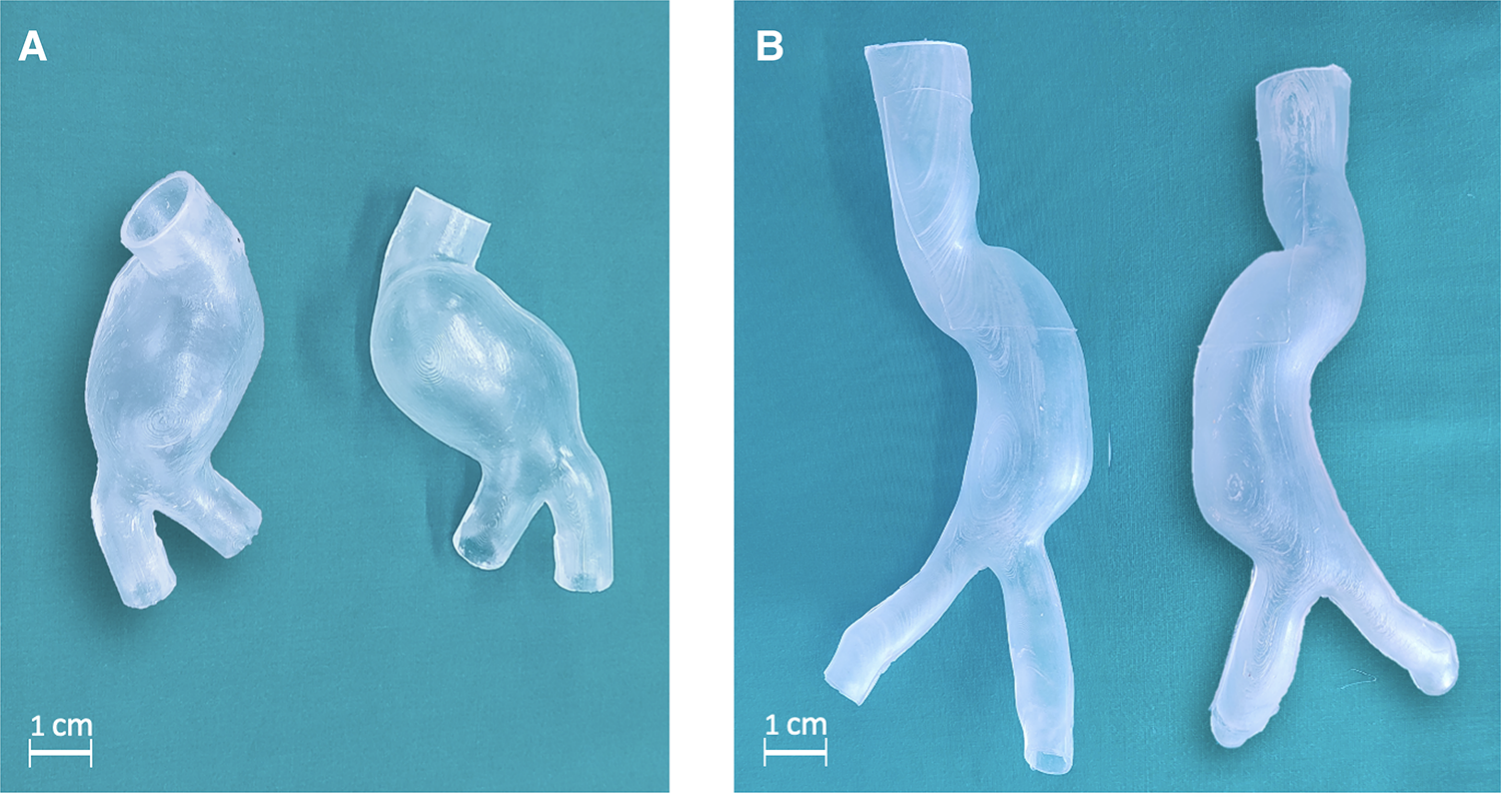
Deformable AAA model obtained through lost molds (**A**) and lost core (**B**) techniques.

The silicone castings succeeded in both *lost molds* and *lost core* casting as shown in Figure 8. The PVA molds dissolved completely without interacting with the casting material. The internal and external surfaces of both silicone models are smooth. Small outgrowths of material are present in the LCM at the joint edges of the three outer molds. This defect is not noticeable in the LMM as the PVA molds allowed for a firmer sealing, and there was no silicone leakage from the outer molds. The main features of the manufactured models are summarized in Table 3, including material costs, necessary equipment, and compatibility with medical imaging modalities.

**Table 3:** Manufacturing features and material costs for AAA models.

	*Lost molds*	*Lost core*
Mold material	PVA	PVA/ABS
Support material	PVA	SSU00
FDM Primary material price	121 €/kg	33.5 €/kg
FDM Support material price	121 €/kg	36.5 €/kg
FDM printing time	22 h	67 h
FDM normalized price	121 €/kg	43.98 €/kg
Cost of molds (Only materials)	27.47 €	27.39 €
Casting material	Sylgard 184	Sylgard 184
Casting material price	277 €/l	277 €/l
Model height	12 cm	26 cm
Casting volume	30 ml	70 ml
3D model cost	8.10 €	19.39 €
Other equipment needed	Vacuum chamber, cleaning tank	Vacuum chamber, cleaning tank
MCL system compatibility	Yes	Yes
PIV compatibility	Yes	Yes
US/XA/CT/MRI compatibility	Yes	Yes

### 3.3. Medical imaging applications and accuracy assessment

**Figure 9:**
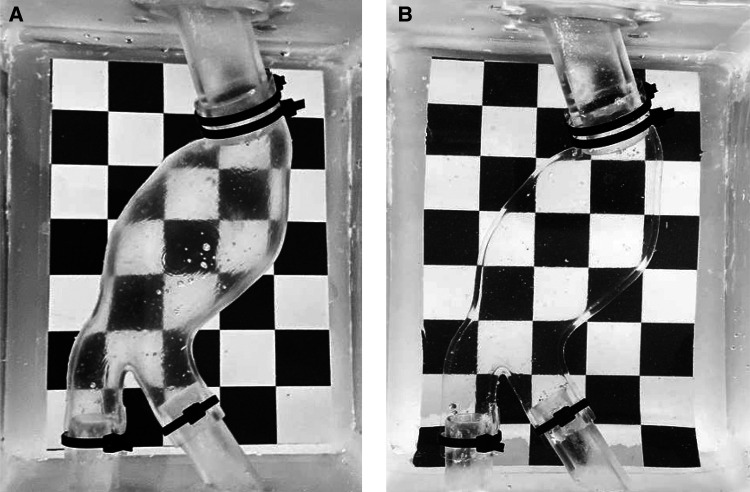
LMM filled with water (**A**) and with a blood-mimicking fluid (**B**).

The manufactured model can be inserted into an MCL system to replicate patient-specific hemodynamic conditions and/or perform PIV investigations. In this case, models are often filled with a blood-mimicking fluid and placed within a purpose-designed box, which may then be filled with water, the same blood-mimicking fluid (in PIV), or gelatin (in US imaging). Figures 9A,B shows LMM immersed in water and a blood-mimicking fluid (mixture of water, glycerol, and urea) that matches the refractive index of Sylgard 184. In the latter case, the models become transparent.

**Figure 10:**
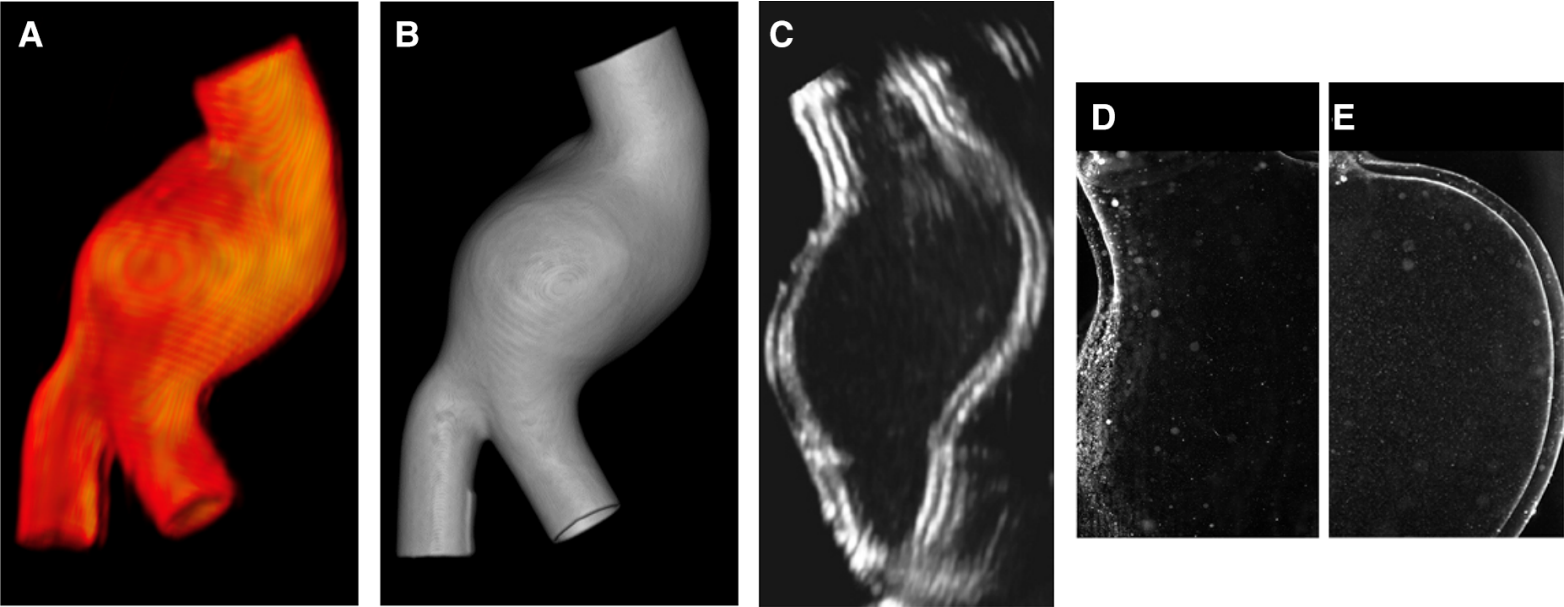
Render of MRI (**A**) and CT (**B**) scans acquired on LMM; B-mode Doppler acquisition in longitudinal view (**C**); PIV images (**D**–**E**).

As reported in Table 3, the models are also compatible with the main medical imaging modalities. Examples of 3D rendering of MRI and CT scans acquired on the LMM are shown in Figures 10A,B, respectively. An example of Doppler B-mode acquisition of the same model in longitudinal view is depicted in Figure 10C. Finally, an example of illuminated particles for PIV is shown in Figures 10D,E. Portions proximal to LMM’s inlet were illuminated to reveal no particle distortion and no light scattering at the solid/liquid interface.

**Figure 11:**
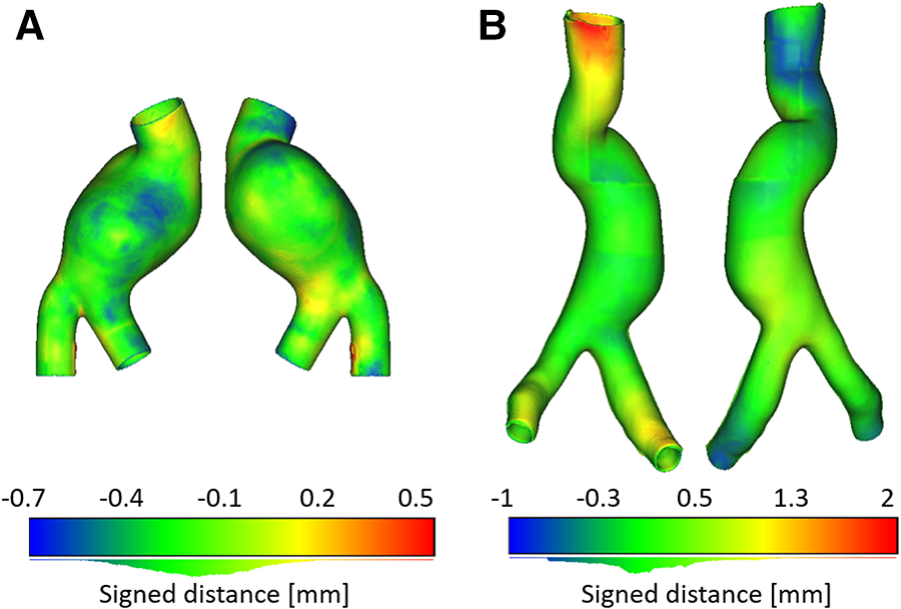
Signed distance [mm] computed for LMM (**A**) and for LCM (**B**). Histograms of distances are depicted below color bars.

CT scans of the two manufactured models were also used to assess the accuracy of the manufacturing with respect to the original design considered as reference. The signed distance between the *manufactured* and *reference* meshes for LMM and LCM is depicted in Figures 11A,B, respectively. This distance takes into account differences in thickness, is expressed in [mm], and is depicted as the spatial distribution of the distance scalar field with the related histograms. Values of signed distance expressed as mean±standard deviation equal to (−0.2±0.2) mm and (0.1±0.6) were computed for LMM and LCM, respectively. In both cases, the regions with the highest distance to the reference are inlets and outlets. This trend is more evident in the LCM and may be caused by a mild misalignment of the core with respect to the outer molds, resulting in wall thickening (red region of Figure 11B) on one side and wall thinning (blue region of Figure 11B) on the other. Nevertheless, these inaccuracies here are not an issue because the AAA models will be plugged into the experimental rig right in these regions.

## 4. Discussion

In the context of the mock-circulatory loop systems, fabricating deformable models represents a crucial step for *in-vitro* quantitative analysis and device testing. This work presented all the phases to manufacture complex patient-specific AAA models with deformable material through *lost molds* and *lost core* casting techniques. In particular, a step-by-step workflow is presented to make the manufacturing of these types of models reproducible and repeatable. In this work, two different geometries have been considered to include two common shapes: a shorter and a longer AAA. However, it is worth highlighting that the presented workflow can be applied to several anatomic configurations, in particular when separation planes are difficult to be defined and/or undercuts are present. Aside from the materials used to print the outer molds and the casting technique, the two models differ in anatomical complexity, size, aneurysmatic bulge shape, and thickness of the final silicone model.

The segmentation of the patient’s images was the first step for manufacturing the patient-specific models. However, this step is user-dependent and can be a source of uncertainty in the resulting 3D-printed model (7). Dealing with patient-specific models, from image segmentation or from statistical shape modelling (54), the CAD phase of the molds necessitated some experience. The complex morphologies presented in this work required the definition of non-conventional separation surfaces, instead of planes, in the mold design phase to ensure correct phantom extraction. Indeed, as many undercuts may occur in such molds, it is not possible to define mold separation from a continuous surface since some regions of the geometry could penetrate the molds during the phantom extraction. In this case, additional separation surfaces must be defined by the user. This aspect and all the phases necessary to identify the separation geometries (including the software commands) and overcome undercuts were described in our work to allow the reproducibility of the fabrication and reduce user dependency.

PVA was chosen due to its hydrosolubility property. However, it is almost four times more expensive than ABS and is highly hygroscopic. For this reason, it was necessary to ensure that molds printed in PVA would not undergo deformation before assembly/material casting caused by the PVA’s sensitivity to humidity. ABS is a plastic material with superior mechanical and thermal properties than conventional plastics, which could also imply greater printing difficulties. Among all the properties, acrylonitrile, butadiene, and styrene provide hardness, rigidity, mechanical strength, impact resistance, and resistance to chemical products to ABS-printed components. ABS can be post-processed (polishing, drilling, gluing, or painting) easily, without special tools or products. Finally, ABS is compatible with silicone. These features determined the choice of ABS to print the outer molds for the LCM. Using ABS instead of PVA for printing outer molds is more advantageous in applications that require many models of the same geometry and is more affordable. In this regard, the FDM printer deposed 623 g of materials for LCM and 227 g for LMM. By normalizing the cost of materials used for FDM for each procedure, the LCM technique costs 43.98 €/kg, including the cost of PVA core, ABS outer molds, and SSU00 supports for printing ABS. The LMM technique costs 121 €/kg as only PVA was used.

A 20% infill and two contours were an acceptable compromise between strength and ease of dissolution of the molds for the LMM. In this case, a fine support density was set for the core to avoid distortions due to PVA hygroscopicity. A 16% infill and 4 contours were instead set to print both outer and core molds for the LCM. Reducing the infill and increasing the contours, silicone had fewer chances to penetrate inside the outer and core, but mold melting was faster now. This parameter variation did not affect the strength and stiffness of the molds. The FDM printer has more nozzles, allowing the simultaneous printing of several materials. For this reason, it was possible to print the supports of the ABS molds in SSU00, allowing for a simpler detachment of the molds from their supports, as well as superior surface quality in the contact regions due to no scratches or roughness following removal. This aspect was important at spots where the molds would come into contact during assembly, such as the gluing channels. The supports of molds in PVA were also printed in PVA through the same nozzle. Using the same material for both molds and supports implied a greater detaching effort.

Manufacturing artifacts and inaccuracies in molds have an impact on the resulting silicone models. In our case, the FDM printer provided an accurate reproduction of the internal and external surfaces of the AAA models (Figures 6 and 7), including a precise replica of the vessel’s curvature at AAA neck and the complex iliac bifurcation in both cases. FDM printers are more affordable than other techniques like laser stereolithography (SLA) printing either for the initial purchase or the used material, printing process costs, and the possibility to print larger volumes. However, SLA would have provided a better surface finish, avoiding the surface roughness typical of the FDM process that required surface treatment. Indeed, surface finish is crucial in determining the outcome of the manufacturing process as surface finish mainly affects the accuracy of measurements or acquisitions in imaging applications.

A careful analysis of casting material was performed to investigate materials able to mimic soft tissues under selected conditions and suitable for imaging applications. The model’s transparency is the most important requirement for PIV, whereas acoustical properties and attenuation coefficient over the diagnostic frequency range (30) are prerequisites of materials in medical ultrasound. Although rigid models could be suitable for flow visualization, the absence of wall compliance could provoke inaccuracies in the hemodynamics and fail in reproducing the actual patient-specific condition. As the result of this analysis, we chose silicone Sylgard 184, a type of silicone rubber commonly used for casting and prototyping applications (49). It is known for its mechanical properties (31, 55), including good tensile strength and elongation. As shown in Figure 10, it is compatible with PIV and other imaging techniques, which can be helpful for pre-surgical planning and research purposes. In addition, Silicone Sylgard 184 has many other useful properties that make it well-suited for use in the fabrication of patient-specific AAA models. These properties include good dimensional stability, low shrinkage, and the ability to replicate fine details. It is also easy to work with and can be easily molded and shaped to create detailed, accurate models with the strength necessary to withstand prolonged physiologically realistic mechanical stresses (27, 55). Additionally, Sylgard 184 can be eventually sealed from exposure to air or water, thus, producing a more stable phantom.

The degassing and casting steps were highly sensitive since any bubble trapped in the material after degassing might harm the outcome. In particular, the presence of bubbles prevents US signal transmission, degrades acquisition quality, and creates scatter artifacts in PIV applications. The use of a controllable vacuum chamber aided in properly degassing the material. It is important to avoid silicone from developing distinguishable layers that would make it difficult to illuminate the various planes for PIV measurements and US acquisitions. Shrinkage can occur during the curing phase, affecting the accuracy of the final model and provoking geometric distortions that affect the model’s flexibility. A major drawback of Sylgard 184 is its tendency to leak out easily from the molds at the junction lines if the molds are not properly sealed. This silicone can also permeate the molds if an adequate number of contours are not set or the contact surfaces are not properly treated. In these regards, the use of the ABS-specific sealant was crucial as was the treatment with PVA glue in the LMM case.

PVA dissolved quickly in warm water (approximately 60/70${}^{\circ }$C) because the printing settings provided for a sufficient trade-off between mold strength and ease of dissolving. Furthermore, the use of an ultrasonic tank sped up the dissolving process, which took three hours for the LMM and up to five hours for the core of the LCM.

The accuracy of the manufactured AAA models, computed in terms of distance to the CAD reference (Figures 11A,B), proved that both casting techniques succeeded in the manufacturing allowing a fine replica of the patient-specific vessels and providing an almost uniform thickness of the final models, with exceptions at inlets and outlets where some wall thickening/thinning occurred especially in the *lost core* case (Figure 11B).

The presented 3D printing techniques minimized the costs as there was no wastefulness of the casting materials. Considering only the materials (PVA, ABS, SSU00, and Sylgard 184), the two AAA models cost € 38.10 and € 46.78, respectively, to which other costs such as, for example, working hours and energy consumed by the FDM printer should be added. In terms of time, the whole manufacturing process from the image segmentation to the final silicone model can be summarized as a couple of days for segmenting the images and designing the molds, up to three days to have the molds printed, up to two days for the preprocessing (surface treatment and drying time, sealing), half a day for preparing the material, degas and cast it, two days to cure, and one day for the post-processing. Finally, we can estimate the duration of the complete process around 11 days.

In this regard, it is worth noting that, if a laboratory has 3D printers, some equipment, and skilled personnel available, the manufacturing process can be more advantageous than buying a model either in terms of cost or time. Buying the LCM from a specialized 3D printing company would have cost roughly € 800 and taken over a month to receive the model. Furthermore, it would always be possible to reproduce the geometry of a specific patient that is particularly useful to better investigate the anatomy and/or the hemodynamics when the *in-vivo* analysis is challenging. It is worth noticing that the patients considered in this work did not present intraluminal thrombi (ILT), and no visceral vessels and/or distal vessels were included. However, there are no specific drawbacks to also include these additional features in the phantoms. Regarding the ILT, the related volume can be included in the outer molds of the model, or it can be manufactured through a dedicated casting procedure and then included in the molds before the material casting by following the approach described in Emendi et al. (56). Regarding the inclusion of additional vessels, such as the renal, splenic, and hepatic arteries, the same process can be followed through the definition of appropriate separation surfaces to avoid undercuts and related problems in demolding the phantom. Indeed, being patient-specific geometries, these AAA models faithfully mimic the behavior of the considered vessel, allowing researchers to capture hemodynamic features as *in-vivo*. In particular, extensive investigations and quantification of hemodynamic features such as velocity, flow patterns, pressures, etc., which play a key role in AAA initiation, development, and potential rupture, can be performed by inserting the AAA models in mock circulatory loop systems (17). Additionally, the AAA models can be used for applications that aim at personalized investigation, such as clinical imaging, surgical training, intervention planning, or therapeutic management that are strictly patient-specific. For instance, a hemodynamic characterization can be performed by processing Color-Doppler images (57) acquired on these models with the noticeable advantage of investigating multiple flow conditions at the inlet, flow splitting at the outlets, and different geometries. However, prospective studies with effective outcomes are beneficial to prove the actual clinical utility of patient-specific AAA models. The accuracy of the manufactured models in representing the patient’s anatomy can be evaluated, possibly by comparing the imaging of the models to the original imaging data. Finally, a comparison between *in-vivo* and *in-vitro* hemodynamic measurements should be performed to verify that *in-vitro* blood flows match *in-vivo* ones.

## 5. Conclusion

Two deformable patient-specific AAA models were fabricated using different techniques, both resulting in accurate replicas made of silicone Sylgard 184 with smooth inner and outer surfaces. Our work aimed at describing the entire phantoms’ fabrication workflow in detail and including all the necessary aspects to allow the reproducibility of the process, thus reducing user dependency. The *lost molds* casting involved printing both the core and outer molds in PVA using an FDM printer and then dissolving the PVA in warm water to create a 1.8 mm thick silicone model. The *lost core* casting technique involved printing the outer molds in ABS using an FDM printer and the core in PVA, which was then dissolved in warm water to create a 2 mm thick silicone model. At the expense of a less smooth surface, the *lost core* casting technique was found to be more cost-effective and allowed for the production of multiple replicas of the same model without having to reprint the outer molds. These models can be used in a variety of applications, including biomedical engineering, training, and hemodynamic investigations in MCL systems.

## Data Availability

The raw data supporting the conclusions of this article will be made available by the authors, without undue reservation.
